# VarioGuide® frameless neuronavigation-guided stereoelectroencephalography in adult epilepsy patients: technique, accuracy and clinical experience

**DOI:** 10.1007/s00701-021-04755-w

**Published:** 2021-02-13

**Authors:** Barbara Ladisich, Lukas Machegger, Alexander Romagna, Herbert Krainz, Jürgen Steinbacher, Markus Leitinger, Gudrun Kalss, Niklas Thon, Eugen Trinka, Peter A. Winkler, Christoph Schwartz

**Affiliations:** 1 Department of Neurosurgery, University Hospital Salzburg, Paracelsus Medical University Salzburg, Ignaz-Harrer-Str. 79, A-5020 Salzburg, Austria; 2 University Institute of Neuroradiology, University Hospital Salzburg, Paracelsus Medical University Salzburg, Ignaz-Harrer-Str. 79, 5020 Salzburg, Austria; 3 Present Address: Department of Neurosurgery, München Klinik Bogenhausen, Englschalkingerstr. 77, 81925 Munich, Germany; 4 Department of Neurology, University Hospital Salzburg, Paracelsus Medical University Salzburg, Ignaz-Harrer-Str. 79, 5020 Salzburg, Austria; 5 Department of Neurosurgery, University Hospital Munich, Ludwig-Maximilians-University Munich, Marchioninistr. 15, 81377 Munich, Germany

**Keywords:** Accuracy, Clinical outcome, Depth electrode, Drug-resistant epilepsy, Epilepsy surgery, Pre-surgical evaluation, SEEG

## Abstract

**Background:**

Stereoelectroencephalography (SEEG) allows the identification of deep-seated seizure foci and determination of the epileptogenic zone (EZ) in drug-resistant epilepsy (DRE) patients. We evaluated the accuracy and treatment-associated morbidity of frameless VarioGuide® (VG) neuronavigation-guided depth electrode (DE) implantations.

**Methods:**

We retrospectively identified all consecutive adult DRE patients, who underwent VG-neuronavigation DE implantations, between March 2013 and April 2019. Clinical data were extracted from the electronic patient charts. An interdisciplinary team agreed upon all treatment decisions. We performed trajectory planning with iPlan® Cranial software and DE implantations with the VG system. Each electrode’s accuracy was assessed at the entry (EP), the centre (CP) and the target point (TP). We conducted correlation analyses to identify factors associated with accuracy.

**Results:**

The study population comprised 17 patients (10 women) with a median age of 32.0 years (range 21.0–54.0). In total, 220 DEs (median length 49.3 mm, range 25.1–93.8) were implanted in 21 SEEG procedures (range 3–16 DEs/surgery). Adequate signals for postoperative SEEG were detected for all but one implanted DEs (99.5%); in 15/17 (88.2%) patients, the EZ was identified and 8/17 (47.1%) eventually underwent focus resection. The mean deviations were 3.2 ± 2.4 mm for EP, 3.0 ± 2.2 mm for CP and 2.7 ± 2.0 mm for TP. One patient suffered from postoperative SEEG-associated morbidity (i.e. conservatively treated delayed bacterial meningitis). No mortality or new neurological deficits were recorded.

**Conclusions:**

The accuracy of VG-SEEG proved sufficient to identify EZ in DRE patients and associated with a good risk-profile. It is a viable and safe alternative to frame-based or robotic systems.

**Supplementary Information:**

The online version contains supplementary material available at 10.1007/s00701-021-04755-w.

## Introduction

Resection of the epileptogenic zone (EZ) remains the treatment of choice in drug-resistant focal epilepsy (DRE) patients [36, 48]. It is quintessential to reliably identify the EZ prior to epilepsy surgery, and stereoelectroencephalography (SEEG) by means of depth electrode (DE) implantation has been established as the “gold standard” for this purpose [3, 6, 11, 30, 34, 39, 54, 55, 57, 62]. Stereoelectroencephalography allows the identification and in-situ evaluation of deep-seated seizure foci and their propagation pathways in DRE patients, for whom non-invasive methods have led to inconclusive or discordant results. In most epilepsy centres, DE implantations are performed by frame-based techniques, or more recently by robotic trajectory guidance systems, which both have been found to result in excellent accuracy [1, 2, 4, 7, 10, 12, 17, 20, 25–28, 32, 38, 40, 43, 47, 49–53, 56, 58, 67]. These techniques, however, are not available in all neurosurgical departments, and frameless neuronavigation-guided SEEG may represent a possible alternative. The VarioGuide® (VG) system (BrainLab AG, Germany) is an already widely used and well-established tool to conduct frameless neuronavigation-guided intracranial biopsies with good accuracy [8, 21, 44, 60]. Data in the context of frameless VG-SEEG are, however, still scarce and to the best of our knowledge, only three studies on VG-SEEG are available [9, 24, 59].

To this end, we share our experiences on VG-SEEG in a series of DRE patients. We focused on an in-depth evaluation of the achieved accuracy, the patients’ outcome and treatment-associated morbidity and complications.

## Methods

### Patients

We retrospectively identified all adult patients, who underwent SEEG for DRE in our interdisciplinary epilepsy centre between March 2013 and April 2019. Informed consent prior to all conducted examinations and surgeries was obtained from all patients/legal guardian(s).

All clinical and surgical data, neurological outcome and treatment-associated morbidity were extracted from the electronic patient charts. Drug-resistant epilepsy was defined by the ILAE in all patients [35]. Pre-surgical evaluations consisted of patients’ history, neurological examination, non-invasive video-EEG-monitoring (VEEG), high-resolution 3 Tesla magnetic resonance imaging (MRI) according to the ILAE protocol, functional MRI for language and memory lateralisation, neuropsychological testing, psychiatric exploration, metabolic imaging (positron emission tomography, ictal single-photon emission computed tomography with SISCOM), magnetoencephalography, electrical/magnetic source imaging (ESI/MSI) and Wada-Test in selected patients [61].

The interdisciplinary epilepsy board including members of the departments of neurology, neurosurgery, neuroradiology, and neuropsychology confirmed the indication for SEEG and subsequent surgical or conservative treatment. Outcome after resective epilepsy surgery was assessed by the Engel Epilepsy Surgery Outcome Scale and the International League Against Epilepsy (ILAE) Classification [22, 23, 64]. Date of last clinical follow-up was April 1, 2020.

### Surgery

An interdisciplinary team, neurosurgeons and epileptologists, conducted the SEEG planning. Preoperatively, patients received an MRI consisting of T1-weighted ± contrast-enhanced (3D-dataset, gadolinium, slice thickness: 1.0 mm), T2-weighted (slice thickness 4 mm) and Fast Field Echo sequences. Data were transferred to the neuronavigation system (BrainLab AG, Germany) and fused to a computed tomography (CT) scan. Trajectory planning was performed via the iPlan® Cranial software Version 3.0 (BrainLab AG, Germany). The trajectories were designed to be as short as possible, not to interfere with vascular structures, not to cross sulci and ventricles. The length of each planned electrode was recorded, and bone thickness was measured on CT at each implantation site.

One neurosurgeon (HK) was involved in all performed surgeons. All DE implantations were conducted under general anaesthesia with the patients’ head fixed in a 3-pin Mayfield® skull clamp (Integra LifeSciences Holding, USA). Then neuronavigation surface matching using the softtouch pointer was conducted, and its accuracy was subsequently verified by anatomical landmarks (e.g. nasion, medial and lateral epicanthus, external auditory canal, scalp surface); a mathematical accuracy of ≤ 1.5 mm (according to the BrainLab navigation algorithm) was deemed acceptable for proceeding with the DE implantations and was achieved in all cases. For the DE implantation, the VG-arm was adjusted according to the selected trajectory and a drill sleeve was inserted. A small 5-mm skin incision and placement of a 2-mm burrhole was conducted, followed by monopolar coagulation of the dura and screwing of a bolt to the bone. The bolt length, either 23 or 31 mm, depended on the bone thickness and presence of temporal muscle. Then a stylet was inserted with a 5-mm distance to the trajectory’s actual target point (TP) in order to prepare the trajectory through the brain parenchyma. Before inserting the electrode, we calculated its length with respect to the depth of the implanted bolt in the bone. For this, the surgeon measured the depth of the bolt in the bone to accurately define the distance between the bolt surface and the TP. The stylet was then removed, and the electrode was inserted via an introducing stylet, which was then also extracted, and the electrode was fixed with a silicone cap. The utilised DEs featured four to 14 contacts and a diameter of 0.86 mm (Ad-Tech® Medical Instrument Corporation, WI, USA).

In three latter cases, electrodes were tested in the operating room to replace electrodes without electrical signals. According to our in-house algorithm, postoperative MRI was routinely performed immediately after each implantation procedure to evaluate electrode positions and to rule out possible haemorrhages. The reasons for this approach are that (1) the post-implantation MRI could be directly used for the planning of potential consecutive neuronavigation-guided focus resections, and (2) we tried to minimise the use ionising radiation in our often young epilepsy patients. The SEEG recordings started immediately thereafter.

### Accuracy assessment and statistical analysis

**Fig. 1:**
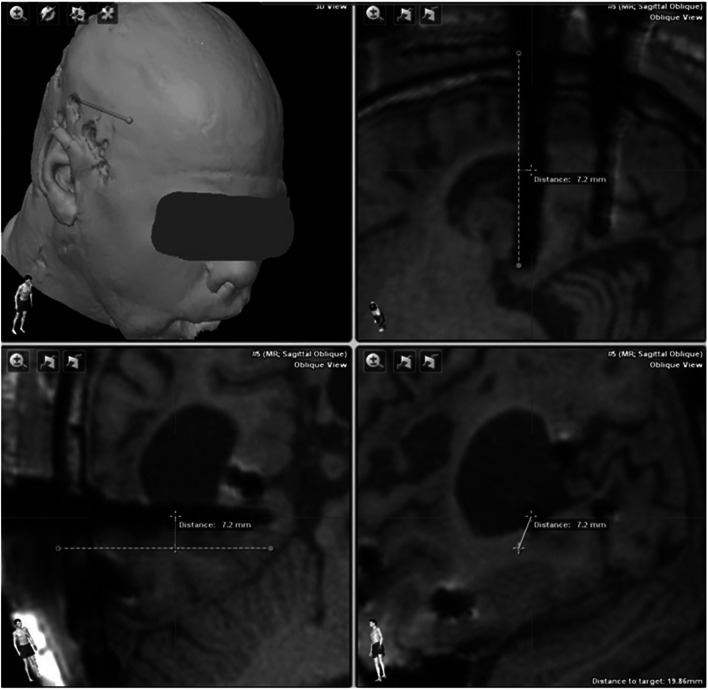
Exemplary accuracy measurement of centre point deviation. The Euclidean distance from the planned preoperative trajectory to the placed electrode in postoperative MRI is demonstrated for the centre point (CP) of a single electrode

Two members of our Neuroradiological Institute (LM, JS) evaluated each patient’s electrodes with regard to their accuracy in comparison to the planned trajectories. The Euclidean distance from the planned preoperative trajectory to the placed electrode in postoperative MRI was measured in Cartesian coordinates. The results had to be transformed into polar coordinates, where the origin of the coordinate system coincides with the individual electrode. This procedure was carried out for each electrode at the entry (EP), the centre (CP) and the target point (TP) of the electrode (Fig. 1). For detailed analysis of deviation, several factors were analysed: the electrode’s (LE), entry angle to the surface of the cortex (EA) and the bone thickness at the EP. To determine the absolute tilt angle of each electrode to the plumb line, the projections of this angle were measured in two different planes, for example in axial and coronal plane. Furthermore, multiple correlation analyses evaluating neuro-anatomical (i.e. bone thickness at EP, implanted lobe) and surgery-related aspects (i.e. EA, DE length) were conducted in an effort to identify factors-associated DE accuracy. The measured accuracy was evaluated as a continuous variable, the EA was assessed as a continuous as well as categorised factor (i.e. ≤ 30° vs. > 30°), and the bone thickness at the EP was analysed as a continuous as well as categorised parameter (i.e. ≤ 10 vs. > 10 mm). The appropriate statistical testing, according to the underlying data format, was performed with SPSS® Statistics version 26 (IBM Software, USA). The level for significance was set to *p* ≤ 0.05.

All accuracy measurements were done in iPlan® Cranial software, and statistical analyses were performed with SPSS® Statistics version 26 (IBM Software, USA).

## Results

### Patients and surgery

The study population comprised 17 consecutive patients (10 women) with a median age at DE implantation of 32.0 years (range: 21.0–54.0). The most commonly implanted locations were the limbic, insular and temporal lobes with a predominance for the right hemisphere (Table 1).

**Table 1:** Patient population and surgery

Patients (*n* = 17)	
Male:female ratio	1:1.4
Mean age (SD) (years)	31.9 (9.3)
Median age (range) (years)	32.0 (20.0–54.0)
Implanted lobes/regions	
Frontal (*n*)	8
Central (*n*)	1
Parietal (*n*)	14
Temporal (*n*)	11
Insular (*n*)	9
Limbic (*n*)	12
Occipital (*n*)	8
Surgeries (*n* = 21)	
Dominant hemisphere implantation (%)	9 (42.9)
Median number of DE/surgery (range)	12.0 (3.0–16.0)
Mean number of DE/surgery (SD)	10.5 (4.1)
Median duration of surgery (range) (min)	196.0 (50.0–360.0)
Mean duration of surgery (SD) (min)	197.3 (59.7)

*DE* depth electrode, *EA* entry angle, *SD* standard deviation

In total, 220 DEs were implanted in 21 SEEG procedures; the median number of implanted electrodes per surgery was 12 (range 3–16) (Table 1). In one patient, an electrode was replaced after intraoperative testing due to an inadequate electrical signal. Postoperative SEEG monitoring was performed for a median time of 12 days (range 3–21). Prophylactic antibiotics were administered for the entire duration of postoperative monitoring.

### Accuracy and safety parameters

**Fig. 2:**
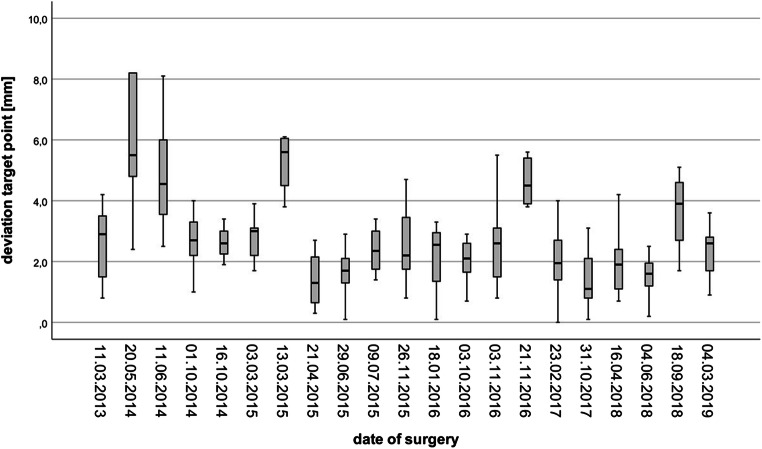
Target point deviations of all performed SEEG procedures

**Fig. 3:**
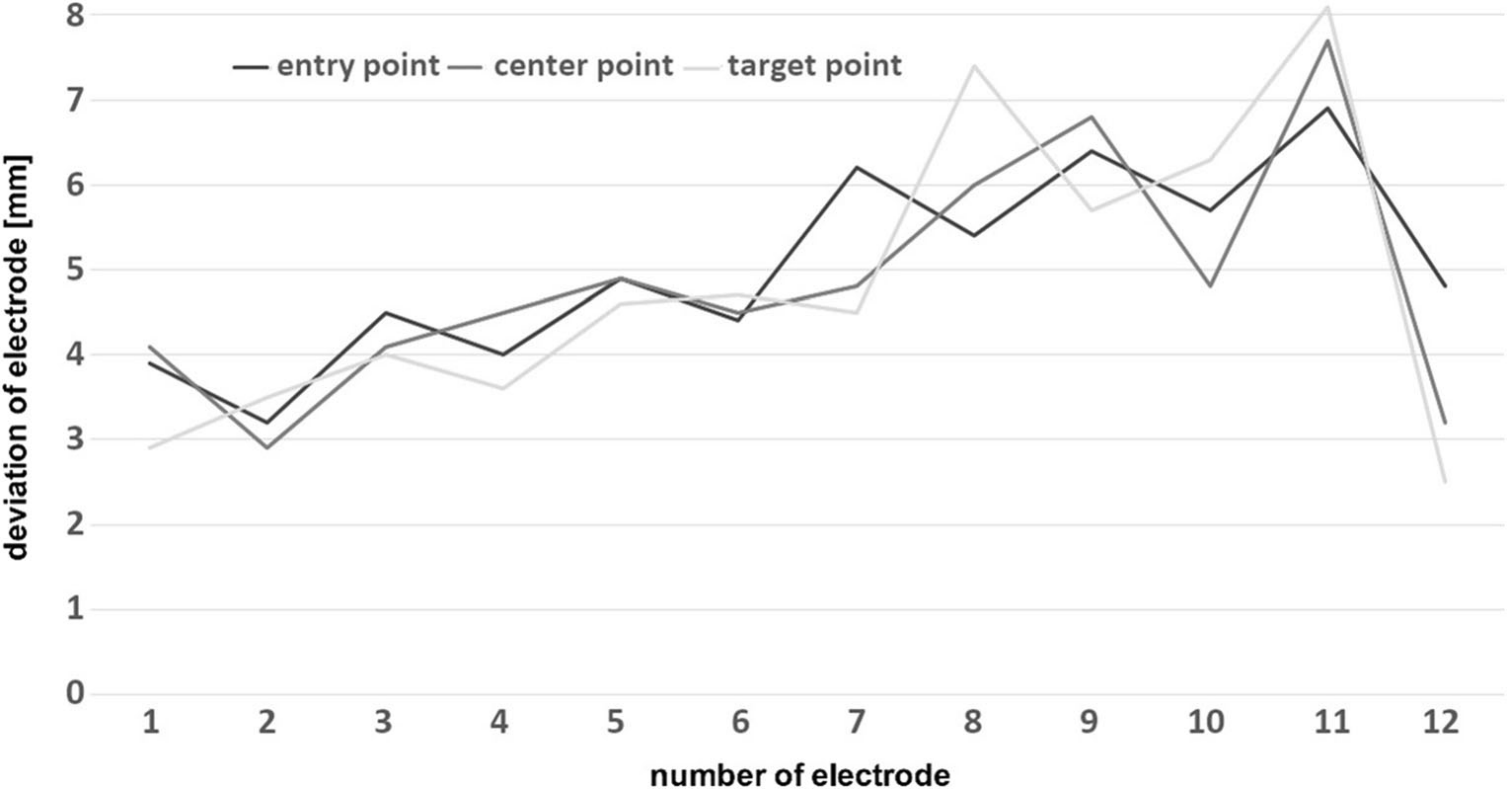
Measured EP, CP and TP deviations for an exemplary patient

The median DE length, measured from the exterior surface of the skull surface to the TP, was 49.3 mm (range 25.1–93.8). The mean deviation in planned versus implanted DE length was 1.9 ± 1.8 mm (Table 2). The mean deviation between the planned trajectory and the actual implanted trajectory, as confirmed by postoperative MRI, was 3.2 ± 2.4 mm for the EP, 3.0 ± 2.2 mm for CP, and 2.7 ± 2.0 mm for TP analyses (Figs. 2 and 3, Table 2). The calculated EA ranged from 1 to 75° (Table 2); 74/220 (33.6%) DEs exceeded an angle of 30°. A bone thickness > 10 mm at EP was recorded for 65/220 (29.5%) DEs. The statistical analyses showed that shorter DE length correlated with worse TP accuracy (*p* = 0.01; Spearman’s correlation); no significant correlations for EA, bone thickness and implanted lobe were recorded (Table 3).

**Table 2:** Accuracy assessment

Implantation and electrode characteristics (*n* = 220)	
Mean DE length (SD) (mm)	49.6 (11.9)
Median DE length (range) (mm)	49.3 (25.1–93.8)
Mean EA (SD) (°)	24.6 (13.3)
Median EA (range) (°)	23.6 (1.0–74.8)
Mean bone thickness (SD) (mm)	9.0 (3.1)
Median bone thickness (range) (mm)	9.0 (2.0–21.0)
Deviation analyses	
Mean EP deviation (SD) mm	3.2 (2.4)
Median EP deviation (range) mm	2.8 (0.1–27.3)
Mean CP deviation (SD) mm	3.0 (2.2)
Median CP deviation (range) mm	2.5 (0.1–25.1)
Mean TP deviation (SD) mm	2.7 (2.0)
Median TP deviation (range) mm	2.4 (0.0–19.4)
Mean ED length deviation (SD) mm	1.9 (1.8)
Median ED length deviation (range) mm	1.6 (0.0–18.4)

*CP* centre point, *DE* depth electrode, *EA* entry angle, *SD* standard deviation, *TP* target point

**Table 3:** Statistical analysis

Correlations—*p* values			
Analysed factor	EP deviation (continuous variable)	CP deviation (continuous variable)	TP deviation (continuous variable)
DE length (continuous variable, Spearman’s correlation)	0.44	0.22	0.01
Entry angle (continuous variable, Spearman’s correlation)	0.84	0.93	0.51
Entry angle (categorized variable, Mann-Whitney *U* test)	0.74	0.46	0.47
Bone thickness (continuous variable, Spearman’s correlation)	0.35	0.68	0.36
Bone thickness (categorised variable, Mann-Whitney-*U* test)	0.77	0.27	0.17
Lobe (categorised variable, Kruskal-Wallis test)	0.20	0.15	0.25

*CP* centre point, *DE* depth electrode, *EP* entry point, *TP* target point

We did not record any SEEG-related new transient or permanent neurological deficits. Of note, no electrode-associated intraparenchymal haemorrhage was seen. For one patient, immediate postoperative MRI revealed a malpositioned DE, which was then promptly corrected within the same anaesthesia. In one patient, a delayed bacterial meningitis requiring prolonged antibiotic treatment was recorded. The recorded SEEG monitoring period of this patient was 20 days. Overall, transient morbidity was seen in 1/17 patients (5.8%); no permanent morbidity or mortality occurred.

### Outcome and treatment-associated morbidity

An adequate signal fidelity was recorded for all but one implanted DEs (99.5%). Overall, in 13/17 patients (76.5%), an EZ was identified by VG-SEEG monitoring. In four cases, however, no reliable EZ localisation could be detected (23.5%). After interdisciplinary consultation, those four patients underwent re-implantations within a period of 10 to 18 days following the initial surgery. Eventually, an EZ was identified in 15/17 (88.2%) patients. Overall, eight of these 15 patients (53.3%) eventually underwent resective epilepsy surgery (Suppl. Table 1). Two patients declined further surgical epilepsy treatment due to personal reasons. In two more patients, the identified EZ was not deemed accessible for safe surgical resection, and three patients were found to suffer from multifocal epilepsy making them no surgical cases (Suppl. Table 1). Within a median clinical follow-up of 40 months (range 2–66), 7/8 (87.5%) of the surgically treated patients experienced a significant reduction of their seizures postoperatively. Six (75.0%) patients were seizure-free (Engel IA, Wieser 1), and another patient experienced one single focal seizure 3 months after surgery, but no further seizures in the course of follow-up (Engel IB, Wieser 3). One patient did not benefit from open epilepsy surgery and underwent vagus nerve stimulator implantation for recurrent seizures (Engel IVB, Wieser 5).

## Discussion

### Main findings

We provide an in-depth analysis of the largest overall number of implanted DEs utilising the VG system. We conclude that (1) even though the accuracy of VG-SEEG is moderately worse than the results obtained by frame-based or robotic-assisted systems, (2) VG-SEEG is associated with a good risk-profile for procedure-related complications, and (3) the attained accuracy is sufficient to reliably identify the EZ. Furthermore, we provide an overview of the current literature (Suppl. Table 2)

### Accuracy, treatment-associated morbidity and outcome

Stereoelectroencephalography techniques are categorised into three distinct technical approaches: frame-based, robot-assisted and frameless surgery (Suppl. Table 2) [1, 2, 4, 5, 7, 9, 10, 12–20, 24–29, 31–33, 37, 38, 40–43, 45–47, 49–54, 58, 59, 65–67]. Superior accuracy is not only important to ensure diagnostic efficacy, but also correlates with surgical complications such as electrode malpositioning and haemorrhage [7, 11, 16, 39, 43, 49, 58]. Frame-based techniques have first been introduced in the early 1960s, and since then constitute the method of choice for stereotactic neurosurgery including SEEG procedures [3, 12, 13, 17–19, 22, 27, 29–31, 33, 35, 36, 58]. Recently, robotic-assisted systems have been introduced enabling excellent accuracy with little periprocedural morbidity in SEEG surgeries [13–15, 20, 23–26, 28, 32, 34, 57, 64]. Not all neurosurgical departments have access to these techniques though. Frame-based systems usually require the involvement of specialised stereotactic neurosurgeons, and robot-assisted systems are still associated with significant costs. In contrast, neuronavigation systems are widely available, and especially the VG system has proven to be a valid alternative to frame-based stereotactic biopsy systems [37–40]. However, it has not been well evaluated in terms of its SEEG capabilities [41–43].

In this context, we hypothesised that VG-assisted SEEG will likely not be able to match the attained accuracy of frame-based/robot-assisted techniques, but might still yield high safety standards and satisfactory diagnostic results allowing minimal-invasive EZ identification [23, 41–43, 48–57, 59–63, 65–67]. Our measured mean TP deviation was 2.7 ± 2.0 mm. With regard to the existing literature, our accuracy results were worse than those achieved by frame-based/robotic-assisted approaches [13, 14, 20, 22–27, 34] Most groups utilising those implantation techniques report TP deviations in the range of 0.3–6.7 mm [14, 20, 22, 24, 26, 34, 36]. In-depth analysis of our data revealed that one patient (Pat. ID 02) showed significantly worse accuracy with mean EP, CP and TP deviations of 8.2 ± 7.7 mm, 8.1 ± 7.1 mm and 7.6 ± 5.4 mm, respectively. Disregarding this specific patient’s results, our recorded mean deviations would have been even better (i.e. mean deviations of 3.0 ± 1.7 mm, 2.8 ± 1.5 mm and 2.5 ± 1.4 mm). Due to the fact that all DEs deviated in the same direction in this case, the most likely explanation was an accidental intraoperative displacement of the neuronavigation marker array. This highlights an important potential pitfall of this neuronavigation-based technique, since the surgeon may not always be aware of marker array movement and discordance between trajectories and neuroanatomical landmarks in a draped patient. To prevent this issue, routinely performed intraoperative recalibrations have now been implemented into our surgical algorithm. In none of the other analysed patients, a comparable deviation in a specific direction (i.e. systematic error) was observed.

We identified electrode length as the only significant influence on achieved accuracy; all other assessed factors did not show significant correlations. The recorded correlation between shorter DE length and worse accuracy was, however, associated with a poor correlation coefficient and must be interpreted with great caution; or, in other words, even longer DEs did not result in worse accuracy. Also, we were not able to confirm prior results suggesting that an unfavourable trajectory EA leads to worse TP accuracy [50]. Nevertheless, we always tried and would definitely recommend to aim for a trajectory EA as perpendicular as possible to the skull surface in order to obtain optimal accuracy. In our surgical setup, we lodged a sharp-teethed drill sleeve onto the calotte in order to minimise slippage. In addition, the positioning of the reference array could also influence accuracy levels. In other words, a larger distance between the reference array and the implantation site could potentially lead to worse accuracy. Thus, special attention to the optimal placement of the reference array should be given during surgical planning.

The main relevance of the procedure’s accuracy is (and therefore main criteria to assess the feasibility of VG-SEEG within the framework of epilepsy surgery), is the surgery-related morbidity and ultimately the patients’ clinical outcome. Naturally, clinical outcome is hereby a reflection of multiple factors comprising patient selection, the SEEG procedure, as well as the interpretation of the obtained data and any potentially resulting epilepsy surgery. Stereoelectroencephalography is generally associated with a low risk for procedure-related morbidity [6, 16]. In our series, we recorded only one SEEG-related complication. Also, we experienced only one single electrode malfunction during the SEEG monitoring period. Utilisable data were obtained for all cases, and an EZ was identified for the vast majority of our patients.

In general, we found the VG-SEEG system to be a feasible and reliable tool for DE implantations. It proved to be rather easy-to-use and, due to the high flexibility of the VG-arm, multiple electrodes may be implanted at different angles and trajectories in one surgery. Our achieved deviation should be considered satisfactory, since we had a low rate of surgical complications and a good detectability of epileptic foci. Also, we found our deviations to be in line with those of other frameless systems (Suppl. Table 2). Moreover, it has to be pointed out that accuracy within the limits of a few millimetres is actually more important with regard to treatment-associated morbidity (i.e. avoidance of blood vessels/critical structures), and actually less so for epileptogenic zone detection since the implantation targets are commonly larger. As a matter of fact, the precise preoperative planning/non-invasive determination of the target area (assumed EZ) is probably much more significant than slight deviations of the implanted DEs.

Another important aspect, which ought not to be overlooked in these financially strained times, are the associated costs. Even though neuronavigation-based systems do come with a significant price tag, they are generally still considerably more affordable than frame-based and, especially, robotic systems.

On first sight, our eventual resection rate after SEEG of ~ 50% may appear to be a disappointing finding, and it is true that compared to the published resection rates of other groups, our percentage of conducted resective treatment is on the lower end (Suppl. Table 2). Even though a significant number of epilepsy patients receive treatment at our hospital, the vast majority of epilepsy patients do not eventually require invasive monitoring. Thus, SEEG is applied rather restrictively and only used for highly selected patients, for whom non-invasive diagnostics and conservative medical treatment did not yield sufficient diagnostic and clinical results. This again might well explain the overall low number of SEEG procedure. Furthermore, even though SEEG was applied for only a highly selected and complex patient cohort, we were able to eventually offer surgical epilepsy treatment options to approximately half of them with good clinical results. As demonstrated by this study, we do believe that VG-SEEG is a safe and reliable tool to identify EZ in DRE patients, and based on these results, we may choose to utilise SEEG more “generously” in future cases.

### Study strengths and limitations

The study’s main strongpoints are the considerable number of implanted DEs and the in-depth accuracy analyses. Experienced neuroradiologists independently performed all measurements and determined not only the TP deviation, but also EP and CP accuracies. The CP analysis, which has not been previously reported, proved that electrodes remained on the trajectories for their entire length. This is of importance, since measurements are not only significant at the TP but also along propagation pathways.

Ultimately, further analyses are needed to establish the accuracy of VG-SEEG. Reported negative results of the correlation analyses may well be attributed to the limited patient number.

## Conclusions

Even though the achieved accuracy by VG-SEEG was moderately worse than results obtained by frame-based or robotic-assisted systems, the technique was associated with a good risk-profile, and proved sufficient to identify the EZ in our sample. Thus, VG-SEEG may be considered a viable and safe alternative for epilepsy centres, which do not have access to frame-based or robotic-assisted implantation technology.

## Supplementary information

Supplementary Table 1 1 Detailed patients’ characteristics (DOCX 22 kb)

Supplementary Table 2 Literature overview (DOCX 34 kb)

## Abbreviations and acronyms

AH: Amygdalohippocampectomy
CP: Centre point
CT: Computed tomography
DE: Depth electrode
DRE: Drug-resistant epilepsy
EA: Entry angle
EP: Entry point
EZ: Epileptogenic zone
ILAE: International League Against Epilepsy
MRI: Magnetic resonance imaging
SEEG: Stereoelectroencephalography
TP: Target point
VG: VarioGuide®
