# Supplementary material for: VarioGuide® frameless neuronavigation-guided stereoelectroencephalography in adult epilepsy patients: technique, accuracy and clinical experience
Source: Acta Neurochir (Wien). 2021 Feb 13;163(5):1355–64. doi: 10.1007/s00701-021-04755-w (PMC8053662; doi:10.1007/s00701-021-04755-w)
Supplement: Supplementary file 1 — 1 Detailed patients’ characteristics (DOCX 22 kb) [file 701_2021_4755_MOESM1_ESM.docx]

***Supplementary Table 1 – Detailed patients’ characteristics***

| **Pat. ID:** | **Sex; Age (yrs):** | **Number of DEs:** | **DE localization:** | **EZ identified:** | **SEEG-related complic-ations:** | **Surgery after SEEG:** | **Outcome (Engel Classification, Wieser):** |
| --- | --- | --- | --- | --- | --- | --- | --- |
| 01 | M; 27 | 9 | Right insular, limbic | Yes | No | Extended AH | IA, 1a |
| 02 | M; 32 | 9 | Left temporal, insular, parietal, frontal | Yes | No | Extended AH | IA, 1a |
| 03 | F; 37 | 12 | Right temporal, parietal, occipital | Yes | No | Focus resection | IA, 1a |
| 04a | F; 23 | 12 | Right temporal, parietal, frontal | No | No | No | No resective surgery performed |
| 04b | F; 23 | 3 | Left temporal | No | No | No | No resective surgery performed |
| 05a | M; 46 | 5 | Right insular, temporal, limbic | Yes | No | Extended AH | IA, 1a |
| 05b | M; 46 | 4 | Right occipital | Yes | No | Extended AH | IA, 1a |
| 06 | M; 37 | 11 | Right occipital, limbic, temporal | Yes | No | Patient denied | No resective surgery performed |
| 07a | F; 54 | 9 | Left insular, parietal, limbic, temporal | Yes | No | Focus resection | IVB, 5 |
| 07b | F; 54 | 4 | Left frontal, parietal, occipital | Yes | No | Focus resection | IVB, 5 |
| 08 | F; 35 | 16 | Left temporal, parietal, frontal, occipital | Yes | Yes (meningitis) | No; eloquent location (i.e. Wernicke's area) | No resective surgery performed |
| 09 | F; 36 | 16 | Right temporal, insular, limbic, frontal, parietal | Yes | No | No; multifocal epilepsy | No resective surgery performed |
| 10 | M; 24 | 12 | Bilateral frontal | No | No | No | No resective surgery performed |
| 11a | F; 27 | 14 | Right temporal, insular, limbic | Yes | No | Focus resection | IA, 1a |
| 11b | F; 27 | 5 | Right occipital | Yes | No | Focus resection | IA, 1a |
| 12 | F; 47 | 14 | Bilateral occipital | Yes | No | No; eloquent location (i.e. optic radiation) | No resective surgery performed |
| 13 | M; 21 | 14 | Right central, parietal, insular, limbic | Yes | No | Focus resection | IA, 1a |
| 14 | F; 38 | 12 | Right parietal, insular, limbic, frontal | Yes | No | Focus resection | IB, 3 |
| 15 | F; 26 | 13 | Left limbic, insular, temporal, frontal | Yes | No | No; multifocal epilepsy | No resective surgery performed |
| 16 | F; 22 | 13 | Left limbic, temporal, occipital, parietal, | Yes | No | No; multifocal epilepsy | No resective surgery performed |
| 17 | M; 28 | 13 | Right occipital, temporal, limbic, parietal | Yes | No | Patient denied | No resective surgery performed |

a,b=Multiple implantations in one patient, AH=Amygdalohippocampectomy, DE=Depth electrode, EZ=Epileptic zone, F=Female, M=Male, SEEG=Stereoelectroencephalography, Yrs=Years
