# Supplementary material for: VarioGuide® frameless neuronavigation-guided stereoelectroencephalography in adult epilepsy patients: technique, accuracy and clinical experience
Source: Acta Neurochir (Wien). 2021 Feb 13;163(5):1355–64. doi: 10.1007/s00701-021-04755-w (PMC8053662; doi:10.1007/s00701-021-04755-w)
Supplement: Supplementary file 2 — Literature overview (DOCX 34 kb) [file 701_2021_4755_MOESM2_ESM.docx]

***Supplementary Table 2 – Literature overview***

| **Authors:** | **Number of patients (n):** | **Methods:** | **Number of electrodes:** | **Overall target point accuracy in mm (range/SD):** | **SEEG-related complications:** | **Number of resective surgeries after SEEG (%):** | **Number of seizure-free patients after resective surgery (%):** |
| --- | --- | --- | --- | --- | --- | --- | --- |
| Abel et al., Journal of Neurosurgery: Pediatrics [1] | 19 vs. 19 | Robot-assisted vs. frame-based in children | 265 vs. 264 | N/A | 2 SDH, 1 ICH | 12 (63) | 4 (33) |
| Abhinav et al., British Journal of Neurosurgery [2] | 29 vs. 5 | Frame-based grid implantation vs. robot-assisted DE implantation | N/A | N/A | 7 hemorrhages and infections | N/A | N/A |
| Afif et al., Operative Neurosurgery [4] | 30 | Frame-based | 35 | N/A | 1 surgical hemorrhage | 27 (90) | 20 (74) |
| Balanescu et al., Stereotactic and Functional Neurosurgery [5] | 4 | Frameless using mT platform stereotactic device | 52 | 1.1 (0.3–1.9) | None | N/A | N/A |
| Bourdillon et al., Journal of Neurosurgery [7] | 50 vs. 50 | Robot-assisted vs. frame-based | 565 vs. 628 | 1.2±0.4 vs. 4.0±1 | 1 pneumatocephalus, 1 SDH | N/A | N/A |
| Budke et al., Operative Neurosurgery [9] | 15 | Varioguide in children | 111 | 3±1.5 (0.6–7.8) | 1 SDH | N/A | N/A |
| Candela-Cantó et al., Acta Neurochirurgica [10] | 14 | Robot-assisted in children | 164 | 1.8 (1.2–2.6) | 1 meningitis, 1 hemorrhage | 11 (79) | N/A |
| Chaitanya et al., Neurosurgical Focus [12] | 24 | Robot-assisted | 182 vs. 153 | N/A | 20.8% hemorrhages | 19 (79) | N/A |
| Chen et al., Stereotactic and Functional Neurosurgery [13] | 7 vs. 11 | Frameless in children comparing surface based vs. refined registration | 81 | 3.1±0.5 vs. 2.2±0.6 | N/A | N/A | N/A |
| D’Agostino et al., Operative Neurosurgery [14] | 13 | Frameless using StarFix microTargeting platform | 180 | 4.9 (3–6.9) | 2 SDH, 1 SAH | 4 (31) | 4 (100) |
| Davies et al., British Journal of Neurosurgery [15] | 12 | Frameless using free hand technique | 15 | 0.8 (0-5) | None | 11 (92) | N/A |
| De Almeida et al., Journal of Neurosurgery [16] | 217 | Frame-based, frameless using FreeGuide and a computer assisted frameless device | 3022 | N/A | 3 abscesses, 3 hematomas | 141 (65) | N/A |
| De Benedictis et al., Neurosurgical Focus [17] | 36 | Robot-assisted in children | 386 | 2.2±1.7 | None | N/A | N/A |
| Dewan et al., Journal of Neurosurgery [18] | 15 | Frameless using StarFix microTargeting platform | 137 | 3.4±2.7 | 4 EDH and SAH | N/A | N/A |
| Dorfer et al., Journal of Neurosurgery [19] | 16 | Robot-assisted | 93 | 1.5 (0.3–6.7) | 1 wound infection | N/A | N/A |
| Dorfer et al., Operative Neurosurgery [20] | 7 | Frameless using bone fixated guide and Vertek articulating arm | 26 | 3.0±1.9 | None | N/A | N/A |
| Fujimoto et al., Neurologia Medicochirurgica (Tokyo) [24] | 7 vs.7 vs. 3 | Frameless comparing 3 different techniques: Free hand vs. frameless stereotactic (Varioguide) vs. CT-guided | 43 | 4.2 (1.5– 8.2) vs. 2.3 (0.9–4.6) vs. 1.1 (1.1–1.4) | 1 bacterial meningitis, 1 hemorrhage | 14 (82) | N/A |
| Gil Robles et al., Stereotactic and Functional Neurosurgery [25] | 9 | Frame-based | N/A | N/A | N/A | 8 (89) | 4 (50) |
| Gonzales-Martinez et al., Epilepsia [26] | 100 | Frame-based | 1310 | N/A | 3 hemorrhages | 75 (75) | N/A |
| Gonzales-Martinez et al., Neurosurgery [27] | 100 | Robot-assisted | 1245 | 1.7 (1.2–2.3) | 3 hemorrhages, 1 surgical hemorrhage | 68 (68) | 45 (66.2) |
| Gonzales-Martinez et al., Neurosurgery [28] | 30 | Frame-based in children | 402 | N/A | 1 ischemic stroke, 1 CSF leak | 18 (60) | 10 (55.5) |
| Gross et al., Journal of Neurosurgery [29] | 62 | Frameless using intraoperative MRI | 413 | 6.2±4.1 | 4.8% infections | 42 (68%) | 26 (62%) |
| Hall et al., Canadian Journal of Neurological Sciences [31] | 53 | Frameless using double-chuck articulated arm (n=20) vs. Robot-assisted (n=33) | 184 vs. 366 | N/A | None | 29 (55) | N/A |
| Iordanou et al., World Neurosurgery [32] | 25 | Robot-assisted | 319 | N/A | N/A | N/A | N/A |
| Kassiri et al., Canadian Journal of Neurological Sciences [33] | 18 | Frameless using Olivier arm | N/A | N/A | None | 18 (100) | 18 (100) |
| Mehta et al., Journal of Neurosurgery [37] | 20 | Frameless using Guide Frame DT and subdural grid implantation | 41 (31 of those via craniotomy) | 3.1±0.5 | 1 SDH | N/A | N/A |
| Mirzayan et al., Stereotactic and Functional Neurosurgery [38] | 87 | Frame-based | 1310 | N/A | 1 hemorrhage | 65 (75) | 40 (62) |
| Munyon et al., Stereotactic and Functional Neurosurgery [40] | 6 | Frame-based and subdural grid implantation | 31 | 1.0±0.2 | None | 3 (50) | 3 (100) |
| Murphy et al., British Journal of Neurosurgery [41] | 13 | Frameless using Guide Frame DT and subdural grid implantation subdural grid implantation | 16 (5 of those via craniotomy) | N/A | 1 osteomyelitis | 13 (100) | 8 (73) |
| Nowell et al., Operative Neurosurgery [42] | 22 | Frameless using Guide Frame DT | 187 | 3.7±2.2 (0–3.4) | 1 hemorrhage | 16 (73) | N/A |
| Ollivier et al., Neurochirurgie [43] | 66 | Robot-assisted | 901 | 2.1 (1.1–3.7) | 1 surgical hemorrhage, 1 stroke-like migraine, 8 hemorrhages | N/A | N/A |
| Rodionov et al., Journal of Neurosurgery [45] | 22 vs.8 vs. 23 | Frameless comparing 3 different referencing techniques: using scalp fiducials visualizing TP vs. bone fiducials visualizing TP vs. fiducials visualizing EP and TP | 187 vs. 83 vs. 202 | 3.8±2.5 to 1.6±0.9 | N/A | N/A | N/A |
| Roessler et al., World Neurosurgery [46] | 6 | Frameless using intraoperative MRI | 58 | 3.2±2.2 (0–8.6) | None | 3 (50) | N/A |
| Rollo et al., Journal of Neurosurgery [47] | 134 | Robot-assisted comparing orthogonal and oblique trajectories | 2040 | 1.7 (orthogonal) vs. 1.9 (oblique) | None | 101 (75) | 55 (54%) |
| Serletis et al., Journal of Neurosurgery [49] | 122 vs. 78 | Frame-based vs. robot-assisted | 2663 | N/A | 5 wound infections, 1 surgical hemorrhage, 1 death | 134 (67) | 61 (68) |
| Sharma et al., Journal of Neurosurgery: Pediatrics [50] | 6 vs. 14 | Frameless vs. robot-assisted in children | 218 | 4.5 (2.8–6.1) vs. 1.1 (0.7–1.6) | None | N/A | N/A |
| Shenai et al., Operative Neurosurgery [51] | 29 | Frame-based comparing 2 referencing techniques | 130 vs. 66 | 3.4±0.9 vs. 4.4±0.9 | None | N/A | N/A |
| Spire et al., Neurosurgical Focus [52] | 4 | Robot-assisted and subdural grid implantation | 7 | N/A | None | 2 (50) | 1 (50) |
| Spyrantis et al., Epilepsy & Behavior [53] | 19 | Robot-assisted, comparing 4 different referencing techniques: CT frame (n=4) vs. CT laser (n=7) vs. 3T MRI (n=7) vs. 1.5T MRI (n=1) | 171 | 2.4 (2.1–2.8) vs.  3.5 (3.1–3.9) vs.  1.7 (1.2–2.2) | 1 ICH | N/A | N/A |
| Stuart et al., Neurosurgical Focus [54] | 1 | Frameless using StarFix microTargeting platform and placement of subdural electrodes | N/A | N/A | None | 1 (100) | 0 (0) |
| Thorsteinsdottir et al., Journal of Neurology [56] | 85 | Frame-based | 160 | N/A | 1 surgical EDH | 72 (85) | 70 (83) |
| Van der Loo et al., Acta Neurochirurgica [58] | 76 | Frame-based | 902 | 2.9 (IQR 2–4.2) | 2.6% major complications | 53 (70) | N/A |
| Verburg et al., World Neurosurgery [59] | 7 | Varioguide | 89 | 3.5 (1.2–13.7) | 1 surgical ICH | 3 (43) | N/A |
| Yeh et al., Journal of Neurosurgery [65] | 16 | Frameless using pylorus system guided by MRI | 96 | N/A | 3 CSF leakages | N/A | N/A |
| Yu et al., World Neurosurgery [66] | 21 | Frameless using StarFix microTargeting platform | 173 | 1.22 | 1 hemorrhage | N/A | N/A |
| Zhang et al., Medicine (Baltimore) [67] | 44 | Frame-based | 364 | N/A | 1 ventricular hematoma | N/A | N/A |
| ***Present study*** | ***17*** | ***Varioguide*** | ***220*** | ***2.7±2.0*** | ***1 bacterial meningitis*** | ***8 (47)*** | ***6 (75)*** |

CSF=Cerebrospinal fluid, EDH=Epidural hematoma, ICH=Intracerebral hemorrhage, MRI=Magnetic resonance imaging, SAH=Subarachnoidal hemorrhage, SDH=Subdural hematoma, TP= Target point
